# Evaluation of a virtual simulation system for root canal irrigation training in preclinical dental education

**DOI:** 10.1186/s12909-025-08387-x

**Published:** 2025-12-03

**Authors:** Xin Yue, Shuai Nie, Yanmei Dai, Jianping Sun, Li Xu, Jing Shen

**Affiliations:** 1 International Dental Clinic, Tianjin Stomatological Hospital, School of Medicine, Nankai University, Tianjin, 300041 China; 2 Department of Preventive Dentistry, Tianjin Stomatological Hospital, School of Medicine, Nankai University, Tianjin, 300041 China; 3 Teaching Office, Tianjin Stomatological Hospital, School of Medicine, Nankai University, Tianjin, 300041 China; 4 College of Artifical Intelligence, Nankai University, Tianjin, 300041 China; 5 Tianjin Key Laboratory of Oral and Maxillofacial Function Reconstruction, Tianjin, 300350 China

**Keywords:** Virtual simulation, Root canal irrigation, Dental education, Endodontic training

## Abstract

**Background:**

Root canal irrigation is a crucial component of endodontic treatment, yet it is often insufficiently addressed in dental education. Although its clinical significance is well recognized, dedicated simulation platforms for irrigation training remain scarce. This study aimed to develop a virtual simulation system for root canal irrigation and to evaluate its effectiveness in improving dental students’ knowledge and procedural competence.

**Methods:**

Thirty-four dental students (26 undergraduates and 8 postgraduates) participated in this prospective study. After receiving standardized theoretical instruction, all students completed baseline and post-training assessments using three-dimensional printed tooth models. Training sessions were conducted with the newly developed virtual simulation system. Outcome measures included theoretical knowledge scores, pre- and post-training practical scores, and simulation-based performance scores. Data were analyzed using paired t-tests, Pearson correlation analyses, subgroup comparisons, and a post-hoc power analysis conducted with G*Power 3.1. In addition, a 26-item Likert-scale questionnaire was administered to assess usability and learner perceptions.

**Results:**

Significant improvements were observed in both theoretical knowledge (mean increase: 0.59 points, *p* < 0.001) and practical performance (mean increase: 3.15 points, *p* < 0.001). Simulation-derived performance scores demonstrated a strong positive correlation with post-training practical outcomes (*r* = 0.73, *p* < 0.001). Questionnaire analysis indicated consistently high ratings for learning effectiveness, usability, instructional value, and overall satisfaction, with Cronbach’s alpha values above 0.80 across all domains.

**Conclusion:**

This virtual simulation system shows preliminary value in improving theoretical and procedural learning in root canal irrigation, with strong acceptability among students. Further validation through larger, multi-center and long-term studies is needed to establish its broader educational impact.

**Supplementary Information:**

The online version contains supplementary material available at 10.1186/s12909-025-08387-x.

## Introduction

Virtual simulation (VS) education, which encompasses virtual reality (VR), augmented reality (AR), and mixed reality (MR) technologies, employs computer-generated three-dimensional models and interactive digital environments to support medical and dental training. By allowing learners to practice procedures outside traditional clinical settings, VS facilitates the development of procedural understanding and technical competency [1]. Owing to its reproducibility, safety, and interactivity, VS has been increasingly integrated into dental education.

Root canal treatment is a cornerstone of endodontic training and comprises several critical stages, including root canal anatomy identification, access cavity preparation, canal instrumentation, irrigation and obturation. In recent years, advances in VS technologies have supported the development of interactive simulation tools for endodontics. Prior studies have demonstrated that immersive VS platforms improve students’ spatial understanding of canal morphology and enhance learning motivation compared with conventional approaches such as two-dimensional radiographs or cone-beam computed tomography [2–4]. Beyond dentistry, numerous international studies have highlighted the effectiveness of simulation-based learning in enhancing self-confidence, satisfaction, and clinical performance among healthcare students. High-fidelity simulation has been shown to promote learner engagement and improve psychomotor and cognitive competencies across various disciplines, including nursing education in Europe and the Middle East [5–8]. These findings collectively underscore the broad pedagogical value of immersive simulation training, supporting its integration into preclinical dental curricula.

Among the procedural components, access cavity preparation has been most extensively standardized within simulation-based training. Commercial platforms, such as the Simodont Dental Trainer, have demonstrated comparable learning outcomes to natural teeth [9] and have been reported to improve fine motor skills and provide realistic haptic feedback [10]. Furthermore, stepwise instructional models that integrate VS with resin blocks or three-dimensional printed teeth have been shown to enhance student performance and confidence [11, 12].

While mechanical preparation is indispensable in root canal therapy, it alone cannot achieve complete microbial elimination due to anatomical complexities. Chemical irrigation, therefore, plays a vital role in ensuring disinfection and treatment success [13]. Despite its importance, preclinical training in irrigation remains largely dependent on extracted human teeth [14]. In China, however, the use of such specimens has been increasingly restricted by ethical regulations [15], leading to reliance on acrylic or three-dimensional printed models [16]. These alternatives improve visibility and standardization but are costly, limited in reusability, and reliant on intensive faculty supervision. More importantly, they fail to reproduce the fluid dynamics of irrigants, restricting both students’ real-time learning and instructors’ ability to evaluate performance.

While virtual simulation tools for endodontic education have continued to evolve, dedicated platforms specifically addressing the irrigation phase of root canal treatment have not yet been widely reported, based on current literature [17, 18]. Existing systems typically cover endodontic procedures such as pulp revascularization [19] or apexogenesis [20] but only superficially address irrigation, often through simplified quizzes or linear task sequences. These approaches lack dynamic interactivity and real-time feedback, both of which are essential for effective procedural learning.

To address these limitations, we developed a virtual simulation system specifically designed for irrigation training in endodontics. The system integrates experimental data from in vitro studies [21] to simulate irrigation fluid dynamics and replicates the clinical workflow through a VS-based interface with real-time performance assessment. This study aimed to evaluate the system’s effectiveness in improving dental students’ theoretical knowledge and procedural skills in root canal irrigation, as well as their overall acceptance of this novel training modality.

In this study, we use the term virtual simulation (VS) as the overarching descriptor for digital simulation–based training technologies. VS encompasses several modalities commonly applied in medical and dental education, including virtual reality (VR), augmented reality (AR), and mixed reality (MR). Although the broader term extended reality (XR) is sometimes used in educational technology literature to collectively refer to VR/AR/MR, the present manuscript consistently adopts VS to avoid terminological overlap and ensure clarity.

## Materials and methods

### Development of the virtual simulation system

The virtual simulation system was independently developed to replicate the workflow of root canal irrigation training. Its design was based on experimental data from prior in vitro irrigation studies [21]. It has been deployed on the Nankai University Virtual Simulation Teaching Platform (https://ilab-x.nankai.edu.cn/#/subject/detail/112), where it is accessible to both internal and external users.

**Fig. 1:**
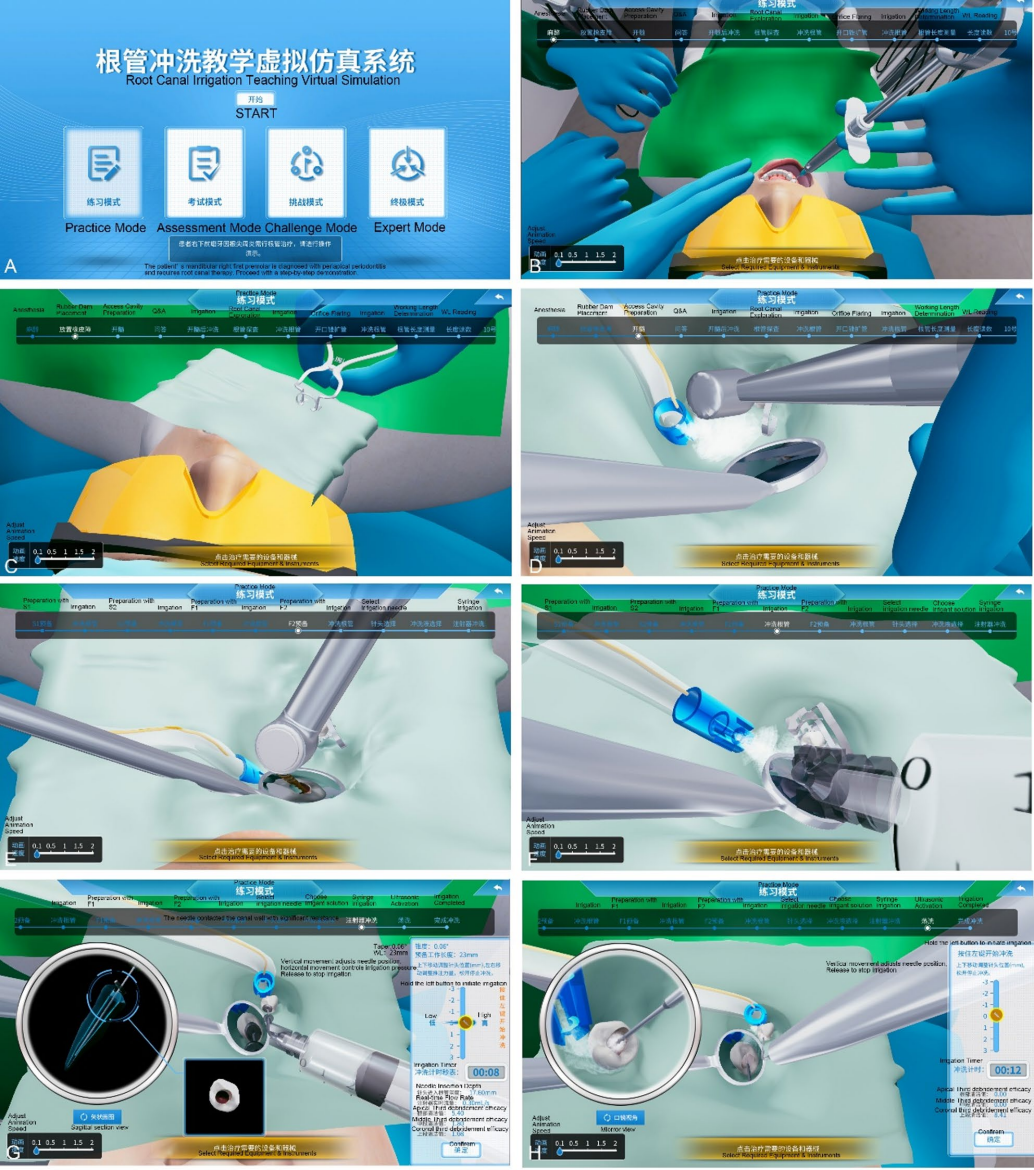
User interface of the virtual simulation system for root canal irrigation training

The platform integrates real-time three-dimensional simulation of irrigant dynamics with a VS interface, providing visual feedback and automated performance evaluation. The simulation encompasses essential procedural steps, including rubber dam isolation, access cavity preparation, canal instrumentation, and a complete irrigation protocol, thereby closely mimicking clinical practice (Fig. 1).

### Training modes

The virtual simulation system provides multiple training modes to support progressive learning and assessment. These include guided learning, practice, and assessment, which allow students to transition from stepwise instruction to independent practice and formal evaluation with automated scoring. In addition, the system incorporates advanced options such as a Challenge Mode and an Expert Mode, which are designed to enhance problem-solving skills and simulate more complex clinical scenarios.

Beyond procedural pathways, the system also integrates quantitative performance indicators, such as a “cleaning index,” to objectively measure irrigation effectiveness and procedural accuracy.

Detailed descriptions of the training modes, operational workflow, and scoring metrics are provided in Additional file 1.

(A) Main menu interface; (B) Preoperative anesthesia module; (C) Rubber dam isolation module; (D) Access cavity preparation module; (E) Root canal shaping module; (F) Irrigation during shaping; (G) Final irrigation using syringe-based technique; (H) Final irrigation using ultrasonic activation.

### Study design

This prospective educational study was conducted at the School of Stomatology, Nankai University, between September 2024 and January 2025. A total of 34 dental students were recruited and stratified into two groups based on academic level:

- *Undergraduate group (n = 26)*: Fourth-year undergraduate students majoring in dentistry at Nankai University.
- *Postgraduate group (n = 8)*: First-year master’s students in dentistry from Nankai University and Tianjin Medical University.

The distribution of participants reflects the typical enrollment structure of the stomatology program, in which undergraduate cohorts include fewer than 30 students and first-year postgraduate cohorts approximately 10. None of the undergraduates had received prior formal instruction in root canal irrigation, whereas all postgraduates had previously studied the topic during their undergraduate curriculum.

### Baseline assessment

At baseline, participants attended a standardized 2-hour theoretical course covering the steps of root canal instrumentation, principles of irrigation, and properties of commonly used irrigants. Each student then performed a complete root canal preparation and irrigation procedure on a three-dimensional printed resin model of a maxillary anterior tooth. The procedures were supervised by four trained teaching assistants, who monitored the accuracy and completeness of each step.

Performance was subsequently evaluated by a blinded experienced endodontist using light microscopy. Cleaning efficacy was assessed with a standardized rubric, and the resulting score was recorded as T1. Participants also completed a theoretical quiz (S1) consisting of eight single-choice questions assessing knowledge of irrigation objectives, irrigant types, mechanisms of action, procedural steps, and complications. Items were stratified by difficulty (three easy, three moderate, two difficult) and selected from a validated question bank routinely used in student examinations.

To ensure consistency in scoring, all examiners completed a calibration process prior to data collection. During this stage, each examiner repeatedly performed the full root canal irrigation procedure following the standardized protocol and evaluated the performances of the other examiners using the same scoring rubric. Through multiple rounds of practice, cross-rating, and discussion, consensus was reached on the interpretation of evaluation criteria, thereby enhancing inter-rater reliability. The scoring rubric was reviewed by senior endodontic educators to confirm its content validity and suitability for preclinical endodontic training.

### Simulation training

Between November 13 and 14, 2024, all participants received standardized training with the Virtual Simulation System for Root Canal Irrigation. Each student completed one session in Practice Mode and one session in Test Mode within a 45-minute session. The system automatically recorded performance metrics, including procedural completion, cleaning efficacy, and a composite score, documented as V1.

### Post-training assessments

To evaluate retention and delayed proficiency, a second practical assessment was conducted one week later, following the principle of the Ebbinghaus forgetting curve [22]. Students repeated the root canal preparation and irrigation procedure on a three-dimensional printed model of a maxillary premolar. The procedures were supervised by the same teaching assistants, and cleaning efficacy was blindly evaluated by the same endodontist. This score was designated as T2.

At the same time, participants completed a second theoretical quiz (S2), designed to be structurally and cognitively equivalent to S1 while avoiding item repetition, to assess changes in theoretical knowledge.

Finally, all students completed a 26-item Likert-scale questionnaire (1 = strongly disagree; 5 = strongly agree) evaluating four dimensions: perceived learning effectiveness, system usability and operation, instructional value and applicability, and overall satisfaction. The questionnaire was adapted from an institutional instrument that has been used for more than a decade, with additional items related to virtual simulation incorporated based on prior VS-based educational studies. Content validity was reviewed by endodontic education experts, and a pilot test was conducted with senior dental students and multiple endodontic faculty members to ensure clarity, relevance, and suitability for formal use.

The complete English version of all questionnaire items is provided in Additional file 3.

**Fig. 2:**
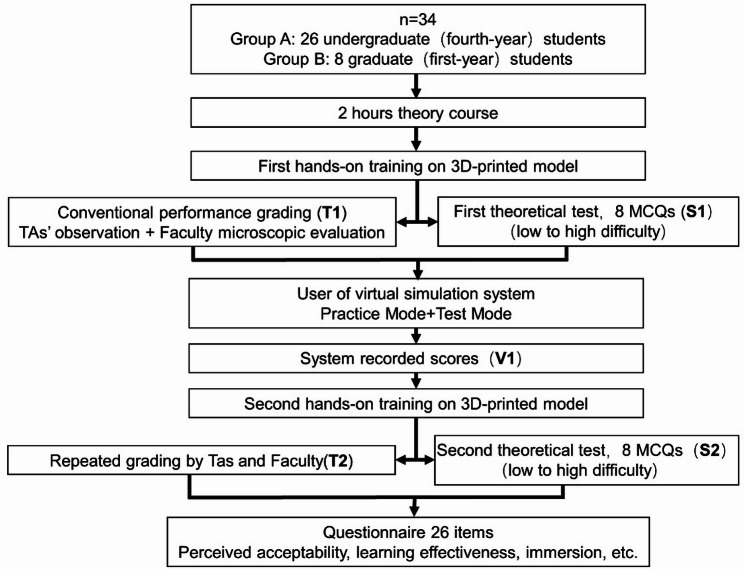
Schematic diagram of the experimental design

An overview of the study design is presented in Fig. 2.

### Statistical analysis methods

A total of 34 participants were enrolled and stratified into an undergraduate group (*n* = 26) and a postgraduate group (*n* = 8). All statistical analyses were performed to evaluate the instructional effectiveness and practical value of the virtual simulation system for root canal irrigation. Data were analyzed using SPSS software, version 20.0 (IBM Corp., Armonk, NY, USA). All tests were two-tailed, and a p-value < 0.05 was considered statistically significant.

Paired-sample t-tests were used to compare pre- and post-training theoretical knowledge scores (S1 vs. S2) and practical performance scores (T1 vs. T2), thereby assessing improvements in both cognitive learning and procedural skills.

Independent-sample t-tests were conducted to compare outcomes between undergraduate and postgraduate groups across multiple domains (S1, S2, T1, T2, and V1), in order to explore the potential influence of academic background on training efficacy.

Pearson correlation coefficients were calculated to examine relationships between virtual simulation performance (V1) and post-training outcomes (T2 and S2). Spearman’s rank correlation was prespecified as a non-parametric alternative; however, inspection of Q–Q plots did not reveal substantial deviations from normality, and Pearson correlations were therefore retained as the primary analytic approach.

In addition, a post-hoc power analysis was performed using G*Power 3.1 (Heinrich Heine University Düsseldorf, Germany) to evaluate the sensitivity of the within-group and between-group comparisons based on the observed effect sizes.

## Results

(The detailed scoring criteria and sample data are provided in Additional file 2.)

### Improvements in theoretical knowledge and procedural skills

Post-training theoretical scores (S2) were significantly higher than pre-training scores (S1), with a mean increase of 9.3% (t(33) = 3.82, *p* < 0.001; Cohen’s d = 0.65). This indicates that the virtual simulation system enhanced participants’ understanding of root canal irrigation.

Practical performance also improved significantly, with a mean increase of 54.4% from T1 to T2 (t(33) = 7.14, *p* < 0.001; Cohen’s d = 1.22), reflecting substantial gains in procedural competence following simulation-based training. Detailed results are presented in Table 1.

**Table 1:** Comparison of pre- and post-training theoretical and practical scores

Assessment	Pre-training (M ± SD)	Post-training (M ± SD)	ΔM (95% CI)	*p*-value
Theory(S1→S2)	6.35 ± 1.15	6.94 ± 0.83	+ 0.59 (0.28–0.90)	< 0.001
Operation(T1→T2)	5.79 ± 3.19	8.94 ± 3.29	+ 3.15 (2.25–4.05)	< 0.001

Paired t-tests were used for all comparisons; df = 33 for all tests

Cohen’s d was 0.65 for theory and 1.22 for operation

### Correlation between virtual simulation scores and learning performance

A strong positive correlation was observed between virtual simulation performance (V1) and post-training practical performance (T2), with *r* = 0.73 (*p* < 0.001), indicating that V1 explained approximately 53% of the variance in T2. The fitted linear regression model was: T2 = 2.84 (± 0.84) + 0.62 (± 0.08, 95% Cl [0.53–0.85], *p* < 0.001) × V1.

Moderate correlations were also identified between V1 and post-training theoretical knowledge scores (S2) (*r* = 0.42, *p* = 0.013), while a weak but statistically significant association was observed between T2 and S2 (*r* = 0.38, *p* = 0.026). These findings suggest that virtual performance not only predicts procedural competence but also partially reflects theoretical knowledge acquisition.

Normality assessments showed no extreme deviations; therefore, Pearson correlation coefficients were used as the primary analytic approach. Detailed correlation coefficients are presented in Table 2.

**Table 2:** Correlation between virtual simulation scores and learning performance

Correlation Pair	*r*	95% CI	*p*-value	*R* ^2^
V1 & T2	0.73	(0.53 to 0.85)	< 0.001	0.53
V1 & S2	0.42	(0.10 to 0.66)	0.013	0.18
T2 & S2	0.38	(0.05 to 0.63)	0.026	0.14

### Post-hoc power analysis

A post-hoc power analysis was conducted to describe the sensitivity of the within-group and between-group comparisons. For the improvement in practical performance (Cohen’s d = 1.13; *n* = 34), the estimated power exceeded 0.99, suggesting high sensitivity for detecting the observed pre–post change. In contrast, the between-group comparison (undergraduates *n* = 26 vs. postgraduates *n* = 8) demonstrated a smaller observed effect size (d = 0.46) and markedly lower power (1 – β = 0.20), reflecting the impact of the small postgraduate subgroup and the uneven distribution. These findings highlight that subgroup comparisons should be interpreted with caution.

### Comparison between undergraduate and postgraduate groups

The postgraduate group consistently achieved higher scores than the undergraduate group across theoretical knowledge (S1, S2), practical performance (T1, T2), and virtual simulation outcomes (V1). However, none of these differences reached statistical significance (all *p* > 0.05; Table 3).

Baseline operational performance (T1) showed a trend toward significance (*p* = 0.096), with a moderate effect size (Cohen’s d = 0.67). While postgraduates demonstrated superior baseline proficiency, the performance gap narrowed after training. The improvement in practical scores was greater among undergraduates (ΔT = 3.30) than postgraduates (ΔT = 2.50), suggesting that the simulation system provided relatively greater benefits for undergraduate students by accelerating the acquisition of procedural skills.

**Table 3:** Comparison of outcomes between undergraduate and postgraduate groups

Variable	Undergraduates (*n* = 26) Mean ± SD	Postgraduates (*n* = 8) Mean ± SD	ΔM (95% CI)	*p*-value
S1	6.15 ± 1.07	7.00 ± 1.20	−0.85 (−1.75 ~ 0.05)	0.063
S2	6.81 ± 0.80	7.38 ± 0.74	−0.57 (−1.18 ~ 0.04)	0.068
T1	5.35 ± 2.84	7.38 ± 3.50	−2.03 (−4.46 ~ 0.40)	0.096
T2	8.65 ± 3.19	9.88 ± 3.44	−1.23 (−3.83 ~ 1.37)	0.345
V1	9.65 ± 3.37	11.00 ± 3.07	−1.35 (−4.05 ~ 1.35)	0.314

Independent-sample t-tests were used for comparisons

Effect sizes (Cohen’s d) were moderate for S1, S2, and T1 (0.67–0.75) and small for T2 and V1 (<0.40). df = 32 for all tests

### Questionnaire evaluation

#### Overall descriptive statistics

Student responses were highly favorable across all dimensions of the questionnaire. Mean ratings exceeded 4.2 on a five-point Likert scale, and more than 90% of responses fell within the 4–5 range, reflecting strong agreement regarding the system’s ease of use, perceived learning effectiveness, instructional value, and overall satisfaction (Table 4). Median scores closely approximated the means, indicating minimal skewness.

**Table 4:** Mean questionnaire ratings by dimension (*N* = 34)

Evaluation Dimension	Mean ± SD	Median
Perceived Learning Effectiveness	4.35 ± 0.78	4.44
System Usability and Operation	4.28 ± 0.82	4.29
Instructional Value and Applicability	4.40 ± 0.75	4.43
Overall Satisfaction	4.45 ± 0.80	4.67

Scores are based on a 5-point Likert scale (1 = strongly disagree; 5 = strongly agree)

Detailed frequency distributions for all items are provided in Additional file 3. Notably, items addressing clarity of step-by-step feedback (Q18) and appropriateness of task difficulty (Q20) received the highest proportions of maximum scores, with more than half of participants selecting “5.” In contrast, the clinical relevance item (Q21) received relatively more neutral responses (14.7%), likely due to limited clinical experience among early-stage trainees.

### Subgroup analysis

Postgraduate students rated all questionnaire dimensions marginally higher than undergraduates, with the largest differences observed in instructional value and overall satisfaction. Mean ratings for both groups exceeded 4.2 on the five-point scale, indicating generally favorable perceptions of the system (Table 5).

**Table 5:** Questionnaire ratings by subgroup

Evaluation Dimension	Undergraduate Students (*n* = 26)		Postgraduate Students (*n* = 8)	
	Mean ± SD	Median	Mean ± SD	Median
Perceived Learning Effectiveness	4.32 ± 0.81	4.33	4.45 ± 0.68	4.56
System Usability and Operation	4.25 ± 0.85	4.29	4.38 ± 0.72	4.43
Instructional Value and Applicability	4.38 ± 0.78	4.43	4.48 ± 0.66	4.57
Overall Satisfaction	4.42 ± 0.83	4.67	4.54 ± 0.71	4.67

Scores are based on a 5-point Likert scale (1 = strongly disagree; 5 = strongly agree)

Undergraduates reported slightly more negative responses regarding system usability, reflected by a higher proportion of low ratings (scores 1–2: 4.2% vs. 1.8% among postgraduates). In particular, Item Q15, which assessed the consistency of virtual hand-motion feedback with user input, received the lowest ratings among undergraduates, with 7.7% assigning a score of 2. This highlights a potential area for further improvement in interactive feedback design.

Postgraduates also demonstrated stronger recognition of the importance of refining irrigation techniques. For example, in response to Item Q23 (perceived need for skill improvement), 75.0% of postgraduates selected the highest score compared with 69.2% of undergraduates.

### Reliability analysis

All questionnaire dimensions demonstrated excellent internal consistency, with Cronbach’s α coefficients exceeding 0.80 across all domains. This result indicates that the items within each dimension were highly reliable in measuring the intended constructs (Table 6).

**Table 6:** Internal consistency of questionnaire domains (Cronbach’s α)

Evaluation Dimension	No. of Items	Total	Undergraduate	Postgraduate
Perceived Learning Effectiveness	9	0.89	0.88	0.85
System Usability and Operation	7	0.87	0.86	0.82
Instructional Value and Applicability	7	0.91	0.90	0.89
Overall Satisfaction	3	0.88	0.87	0.84

## Discussion

This study evaluated the educational effectiveness of a self-developed virtual simulation system specifically designed for root canal irrigation training in preclinical dental education. The findings demonstrated significant improvements in both theoretical knowledge and procedural skills, and student feedback indicated high acceptance and satisfaction. Together, these results support the system’s potential integration into competency-based endodontic curricula.

### Enhancements in theoretical knowledge and procedural competence

Post-training assessments revealed significant gains in theoretical knowledge and operational proficiency. Procedural performance showed a substantial improvement, and theoretical knowledge also demonstrated meaningful gains, supporting the effectiveness of the simulation in enhancing both psychomotor and cognitive competencies. These results underscore the value of the simulation in developing psychomotor and cognitive competencies. Features such as sagittal-view visualization and real-time feedback likely contributed to these outcomes by making otherwise “invisible” irrigation dynamics more accessible to learners. These findings are consistent with earlier research demonstrating that simulation-based training supports procedural mastery and conceptual understanding in dentistry [2, 11, 23].

### Correlation between virtual scores and learning outcomes

A strong correlation was observed between simulation-derived performance scores (V1) and post-training practical performance (T2) suggesting that simulation metrics may serve as meaningful indicators of student competence. Similar observations have been reported in prior work showing that virtual training outcomes can reflect clinical readiness [25]. A moderate correlation between V1 and theoretical scores further suggests that simulation contributes to both procedural and cognitive development.

### Differential learning outcomes across academic levels

Although postgraduates scored higher across most domains, none of the intergroup differences reached statistical significance. Notably, undergraduates demonstrated greater relative improvement in practical performance, suggesting that less experienced learners may derive greater benefit from simulation-based instruction. Subgroup analysis further indicated that undergraduates valued feedback mechanisms and visual guidance more highly, whereas postgraduates highlighted the need for greater task difficulty and more realistic haptic features. These findings align with prior studies suggesting that learners at different stages require tailored simulation designs [24].

It is also important to consider that postgraduate students likely entered the study with higher baseline knowledge and more extensive preclinical exposure, which may act as a confounding factor even in the absence of statistically significant intergroup differences. Their prior familiarity with endodontic principles could partly account for their higher absolute scores and comparatively smaller performance gains. Conversely, the greater relative improvement observed among undergraduates may reflect the larger learning margin typical of novice learners. Future research with stratified sampling, baseline competency matching, or randomized group allocation will be essential to more precisely isolate the independent contribution of the virtual simulation system and reduce potential bias introduced by prior experience.

### Student acceptance and system usability

Survey responses indicated high overall satisfaction across all evaluated domains, and internal consistency analyses further supported the reliability of the questionnaire. Students particularly emphasized the value of stepwise feedback and progressive task difficulty. However, the relatively higher proportion of neutral responses to clinical relevance suggests that future iterations could incorporate case-based or clinically contextualized scenarios to strengthen applicability for preclinical learners.

### Innovations and educational implications

Compared with existing virtual simulation tools that focus primarily on root canal anatomy or access cavity preparation, the present system uniquely targets the irrigation phase—an essential yet underrepresented component of endodontic training. Through the integration of immersive visualization, interactive procedural guidance, and quantitative scoring, the system facilitates both skill acquisition and objective assessment.

Drawing on the current findings, we propose a three-stage instructional framework that incorporates the system into preclinical curricula:

#### Theoretical instruction → Virtual simulation training → Phantom-head practice

Such an approach has the potential to strengthen psychomotor integration, enhance procedural fluency, and support competency development in preparation for clinical practice.

The system’s automated feedback, reproducibility, and standardized evaluation metrics align well with blended and competency-based educational models. It offers scalable training opportunities that reduce faculty burden while enabling monitoring of learner progress across multiple performance dimensions.

A brief comparison with commercial simulators further clarifies its educational positioning. Simulators such as Simodont^®^ provide high-quality visual–tactile experiences for access cavity preparation and caries removal but do not incorporate irrigation-specific training modules. In contrast, the present system focuses exclusively on the irrigation phase and includes real-time visualization and procedure-specific performance scoring. Because it operates on standard computer hardware rather than specialized haptic devices, it can be readily implemented across institutions with varying resource levels, serving as a complementary tool within the broader ecosystem of dental simulation technologies.

### Limitations and future directions

This study has several limitations that should be considered when interpreting the findings. First, the overall sample size was modest, and the distribution between academic levels was uneven (26 undergraduates vs. 8 postgraduates). As indicated by the post-hoc power analysis, the between-group comparison was markedly underpowered despite the large effect size observed for the pre–post improvement. Consequently, subgroup comparisons have limited statistical sensitivity and generalizability, and their interpretation should remain cautious. Larger and more balanced cohorts, preferably across multiple institutions, will be required to validate these preliminary observations.

Second, the study employed a single-group pre–post design without a control group. As such, the independent contribution of the virtual simulation system cannot be isolated from general learning progression or repeated exposure effects. Future studies should adopt randomized controlled or parallel-group designs to more rigorously evaluate incremental educational benefits relative to conventional instruction.

Third, although theoretical assessments used randomized item banks and all participants were first-time learners of both the theoretical content and the simulation-based procedure, potential testing effects cannot be fully excluded. The practical assessments required repetition of the same standardized workflow, which may introduce procedural familiarity. Incorporating parallel-form assessments, delayed post-tests, and OSCE-based evaluations will help clarify long-term retention and clinical transferability.

Fourth, although examiner calibration was performed and standardized rubrics were used, some degree of subjectivity is inherent in performance scoring. Integration of AI-assisted scoring and automated error-tracking algorithms may further strengthen reliability in future work.

Fifth, the questionnaire was adapted from an established institutional instrument and demonstrated good internal consistency, yet response biases such as acquiescence or social desirability cannot be entirely ruled out, particularly given the lack of negatively keyed items. Additional psychometric validation, including factor analysis and response-bias diagnostics, is warranted in subsequent studies.

Finally, this was a single-institution study conducted within a specific curricular structure and student population, which may limit external generalizability. Differences in prior knowledge, particularly between undergraduate and postgraduate learners, may also introduce unmeasured confounding. Multi-center studies with larger and more diverse cohorts, along with examination of the system’s role within blended and competency-based curricula, will help determine its broader applicability. Future versions of the platform should also iteratively refine system features based on user feedback, including enhanced scenario complexity, adaptive difficulty modes, and improved haptic realism.

## Conclusion

This study provides preliminary evidence supporting the educational usefulness of a virtual simulation system specifically designed for root canal irrigation training. The system demonstrated positive effects on both theoretical knowledge and procedural skills, with undergraduate students showing particularly substantial improvement. The significant association between simulation-derived performance metrics and practical outcomes suggests potential value for formative assessment and structured feedback in preclinical endodontic education.

High levels of learner satisfaction also indicate good acceptability and perceived educational value. However, we acknowledge that the present findings represent initial validation only, and comprehensive psychometric evaluation—together with multi-center studies, expanded scenario development, and long-term follow-up—will be required to fully establish the system’s reliability, generalizability, and curricular impact.

By addressing a procedural stage that remains underrepresented in current simulation platforms, this irrigation-focused system offers a scalable tool that may contribute to competency-based dental training when supported by future evidence.

## Supplementary Information

Supplementary Material 1. Additional files 1: Detailed descriptions of the training modes, operational workflow, and scoring metrics.

Supplementary Material 2. Additional files 2: Scoring criteria and raw test results.

Supplementary Material 3. Additional files 3: Frequency distribution of questionnaire responses.

## Data Availability

The datasets generated and analyzed during the current study are available from the corresponding author on reasonable request.
