# Supplementary material for: Evaluation of a virtual simulation system for root canal irrigation training in preclinical dental education
Source: BMC Med Educ. 2025 Dec 3;26:27. doi: 10.1186/s12909-025-08387-x (PMC12781241; doi:10.1186/s12909-025-08387-x)
Supplement: Supplementary file 1 — Supplementary Material 1. Additional files 1: Detailed descriptions of the training modes, operational workflow, and scoring metrics. [file 12909_2025_8387_MOESM1_ESM.docx]

**Additional file 1: Detailed descriptions of the training modes, operational workflow, and scoring metrics**

**1. Introduction to the Virtual Simulation System for Root Canal Irrigation**

The virtual simulation system was developed on the Unity engine and integrates multimedia visualization, interactive computing, and web-based functionality. Training content is presented in a three-dimensional interface accessible on a computer screen, allowing students to independently log in and complete the modules online. The system can also be accessed through dedicated VR devices to provide an immersive training experience.

Four training modes are available, arranged in ascending order of complexity: **Practice Mode, Test Mode, Challenge Mode, and Expert Mode**. Depending on the selected mode, learners may encounter up to 41 interactive decision points, respond to 7 theoretical questions, and complete a full virtual root canal irrigation procedure. Each procedural step is scored independently. Real-time feedback and operation guidance are available in Practice Mode to facilitate learning and error correction.

The platform supports repeated practice, individualized procedural pathways, and quantitative performance feedback. Instructors can monitor learner activity and performance metrics through a backend management interface. No restrictions are placed on usage frequency, and the platform is also accessible to registered users outside Nankai University. Several system parameters—including irrigant flow rate, fluid distribution, and simulated bacterial clearance—were modeled based on prior in vitro experimental data generated by the research team.

**2. Structure and Functional Design of the Virtual Simulation System for Root Canal Irrigation**

The system provides four progressively structured training modes that allow learners to transition from guided practice to advanced clinical scenarios. Each mode differs in terms of instructional support, case complexity, and performance feedback.

**2.1 Training Modes**

The main interface of the system offers four progressively structured training modes:

(1) **Practice Mode**: Designed for beginners, this mode offers step-by-step guidance with visual prompts and real-time error correction. Each procedural step must be completed correctly before moving forward, ensuring mastery of basic skills.

(2) **Test Mode**: Intended as an assessment environment, this mode disables all prompts and guidance. Students are required to complete the irrigation procedure independently, and scores are automatically generated based on accuracy, efficiency, and adherence to protocol.

(3) **Challenge Mode**: Adds clinical variability by introducing common complications, including narrow canals, open apices, severe root curvature, and persistent infection. Additional irrigants (such as 2% chlorhexidine) and activation devices (such as sonic agitation systems) are available, requiring learners to apply judgment in selecting appropriate strategies.

(4) **Expert Mode**: Represents the highest level of difficulty by randomly combining two complications from Challenge Mode, thereby simulating multifactorial clinical scenarios. This mode aims to train adaptability, decision-making, and advanced problem-solving.

A comparative overview of the four training modes, including objectives, key features, feedback mechanisms, and difficulty levels, is provided in Supplementary Table 1.

**Supplementary Table 1. Comparison of training modes in the virtual simulation system for root canal irrigation**

| **Training mode** | **Main objective** | **Key features** | **Feedback mechanism** | **Difficulty level** |
| --- | --- | --- | --- | --- |
| Practice Mode | Stepwise familiarization with the procedure | Guided learning with prompts and visual aids | Real-time guidance and corrective feedback | Low |
| Test Mode | Independent completion of the full irrigation protocol | Students complete tasks without prompts | Automated scoring based on accuracy, efficiency, and adherence to protocol | Moderate |
| Challenge Mode | Development of advanced skills under more demanding conditions | Time limits, increased decision points, and stricter scoring criteria | Limited feedback; scores emphasize precision and efficiency | High |
| Expert Mode | Simulation of clinical-level performance | Complex scenarios requiring integration of knowledge and skills | Summative scoring only; no real-time feedback | Very high |

**2.2 Operational Workflow Overview**

In Practice Mode, learners initiate the simulation by entering a virtual operatory environment. The procedure is guided by an on-screen workflow prompt that outlines the standard sequence of clinical steps for root canal preparation (Supplementary Figure 2A). When incorrect instruments or procedural steps are selected, the system provides immediate corrective feedback. For example, attempting to perform access cavity preparation without prior administration of local anesthesia prompts a warning message, requiring learners to rectify the error before proceeding.

In Test Mode and Challenge Mode, instructional feedback is minimized and appears only in response to critical errors that prevent further progression. Each procedural step is scored independently: minor errors result in point deductions, while major errors—such as irrigant extrusion beyond the apex or persistent file binding—lead to immediate termination of the simulation.

A detailed schematic of the complete operational workflow is presented in **Supplementary Figure 1.**

**2.3 Root Canal Preparation Module**

The first module incorporates local anesthesia, rubber dam isolation, access cavity preparation, and root canal shaping. It is designed to reinforce learners’ familiarity with the clinical workflow and the correct sequencing of endodontic instruments. Each procedure is presented through animated visualizations, with adjustable playback speeds to accommodate different learning preferences. A mid-procedure theoretical quiz is integrated to assess understanding of irrigation principles (Supplementary Figure 2B).

The operational protocol is based on the single-length technique using the ProTaper Gold system (Dentsply, USA), which is widely adopted in undergraduate endodontic training in China. This module emphasizes the principle that irrigation should be integrated throughout the entire canal preparation process. After each file change, learners are required to select an appropriate irrigation needle and perform irrigation accordingly.

To replicate clinical conditions more closely, a dedicated submodule for working length determination was developed. The system simulates the operation of an electronic apex locator with a corresponding monitor interface (Supplementary Figure 2C). A randomized true working length is internally assigned and concealed from the user. Learners must interpret the rubber stop position on the endodontic file and manually input the measured value into the system (Supplementary Figure 2D). Significant deviations from the actual working length result in penalties in the subsequent performance score.

**2.4 Final Irrigation Module**

The second module focuses on final irrigation and emphasizes the standardized implementation of irrigation protocols. Learners are required to make sequential selections at each decision point:

- Irrigation method – conventional syringe irrigation or ultrasonic activation.
- Needle type and port configuration (Supplementary Figure 2E).
- Irrigant parameters – including type, concentration, and drawn volume.
- Insertion depth of the irrigation device within the canal (Supplementary Figure 2F).
- Visualization mode – mirror view or sagittal section (note: only mirror view is available in non-practice modes).
- Irrigant delivery controls – learners regulate flow rate and simulate vertical needle motion through interactive commands (Figures 1G–H).

The system continuously calculates the spatial relationship between the irrigant device (needle or ultrasonic tip) and the root canal anatomy. Inaccurate working length measurements, excessive force, or improper insertion angles trigger the simulation of clinical complications, such as instrument binding or apical extrusion. In Practice Mode, these errors generate immediate corrective feedback (Supplementary Figure 2G). In addition, the module enforces a minimum irrigation volume of 20 mL; the procedure cannot be completed unless this requirement is fulfilled.

**2.5 Cleaning Index and Efficacy Evaluation**

The system integrates a quantitative Cleaning Index, calculated independently for the coronal, middle, and apical thirds of the root canal. Scores are generated based on multiple parameters, including irrigant type, flow rate, needle configuration, and insertion depth.

In Challenge Mode and Expert Mode, the system incorporates a simulated resistant intracanal infection. Effective debridement in these scenarios requires the application of advanced irrigation protocols, such as the sequential use of EDTA and sodium hypochlorite (NaOCl) or the activation of irrigants with ultrasonic devices.

Final irrigation efficacy is determined by the cumulative Cleaning Index across all canal segments, providing an integrated measure of procedural performance.

**2.6 Final Scoring and Feedback Mechanism**

In the operational workflow described in Additional file 1, each procedural step is configured with weighted decision points. Each selectable option corresponds to a predefined score determined by its clinical relevance and appropriateness. The final performance score is composed of three primary components:

(1) Root canal preparation score: Derived from Module 1, this component evaluates the learner’s execution of sequential tasks, including preoperative local anesthesia, rubber dam isolation, access cavity preparation, and canal instrumentation.

(2) Performance score for final irrigation execution – based on correct selection of irrigation method, needle configuration, insertion depth, and delivery parameters.

(3) Irrigation efficacy score – derived from the Cleaning Index, calculated independently for coronal, middle, and apical canal thirds

The system provides both numerical outputs and categorical ratings (e.g., excellent, satisfactory, needs improvement) to deliver objective and quantitative feedback on learner performance (Supplementary Figure 2H). This dual evaluation framework facilitates individualized learning by enabling students to identify strengths and weaknesses.

**3. Scoring Rubric of the Virtual Simulation System**

In the operational workflow outlined, each procedural step within the simulation system is configured with weighted decision points. Each selectable option corresponds to a specific score determined by its clinical relevance and appropriateness. The final performance score is composed of three primary components:

(1) Root Canal Preparation Score

Derived from Module 1 (Supplementary Figure 1), this score assesses the learner’s execution of sequential tasks including preoperative local anesthesia, rubber dam isolation, access cavity preparation, and root canal instrumentation.

(2) Root Canal Irrigation Technique Score

Derived from Module 2, this score reflects performance during the final irrigation stage. Parameters include instrument selection and handling, irrigant sequence, flow rate control, and insertion depth of the irrigation needle.

(3) Irrigation Efficacy Score

Based primarily on the Cleaning Index, this component quantifies the cleanliness of the canal walls. It also incorporates the appropriateness of the overall irrigation strategy, including the sequence of irrigant application, type and concentration selected, delivery technique, total irrigant volume, and avoidance of procedural errors.

For example, direct application of sodium hypochlorite (NaOCl) following chlorhexidine (CHX) irrigation will result in score penalties due to the risk of precipitate formation. In such cases, the maximum attainable efficacy score will be capped at 50% of the default limit.

**3.1 Cleaning Index Evaluation**

The root canal system is segmented into three zones for independent scoring:

- Apical third: the apical 3 mm
- Middle third: the next 4 mm segment
- Coronal third: the remaining coronal portion of the canal

Each zone has a maximum Cleaning Index of 100 points, determined by algorithmic models validated through prior experimental datasets.

**3.1.1 Cleaning Index Value Algorithms**

(1) Syringe Irrigation

Let x represent irrigation time (seconds), and y the Cleaning Index value. At a maximum flow rate of 0.5 mL/s, the algorithm is defined as:

0<x<10, y = arctan(x) × (0.0005x^3^+ 0.01x^2^+ 3.5)

10≤x<20, y = arctan(x) × (0.005x^2^ + 0.185x + 2.35)

20≤x<40, y = arctan(x) × (7.076e^(–0.227[x–20]) + 0.924)

40<x, y = arctan(x)

(2) Ultrasonic Activation

Let z denote activation time (in seconds):

z≥12, y = 1 / (x – 10)^2^ + 0.05

z<12, y = log_10_ (14 – x)

Graphical representations of the Cleaning Index algorithms are shown in **Supplementary Figure 3**.

**3.1.2 Needle design and efficacy zones**

The needle type determines the effective cleaning range. For instance, side-vented irrigation needles are assumed to be effective in the 1.5 mm zone apical to the tip. Cleaning Index score allocation follows these rules:

- Apical irrigation: Middle third receives 1/3 of the apical score; Coronal third receives 1/5 of the apical score.
- Middle third irrigation: Coronal third receives 1/2 of the middle score; Apical third receives 0.
- Coronal irrigation: No contribution to apical or middle thirds.

**3.1.3 Challenge and Expert modes – special infection handling**

In Challenge and Expert modes simulating severe infections, the system includes "special infection" zones (e.g., biofilms) that require advanced strategies for removal. Valid methods include:

- EDTA irrigation followed by NaOCl, separated by an intermediate rinse
- CHX irrigation followed by NaOCl, with a rinse in between
- Ultrasonic activation of irrigants
- High-concentration NaOCl delivery with a 1-minute static soaking phase (requiring operation pause)

Completion of any of these qualifies for both the standard Cleaning Index and an additional bonus score, denoted as cleaning Index β. The maximum cleaning Index β value per canal segment is 50 points.

**3.2 Final irrigation efficacy score calculation**

The total irrigation efficacy score for each canal is calculated as:

Final Irrigation Efficacy Score = (Apical Segment Score) + (Middle Segment Score × 0.5) + (Coronal Segment Score × 0.2)

This weighted calculation emphasizes the clinical importance of apical and middle canal cleanliness in endodontic success.

**3.3 Performance score classification criteria**

Each module stage is individually rated as Grade S, A, B, or C based on the achieved performance score, corresponding to weighted values of 8, 4, 2, and 1 point(s), respectively (**Supplementary Table 2**).

**Supplementary Table 2 Performance score classification criteria for different simulation modes**

| **Mode** | **Grade** | **Preparation Score** | **Irrigation Technique Score** | **Irrigation Efficacy Score** |
| --- | --- | --- | --- | --- |
| Practice & Exam Mode | S | [39-48] | [30-37.6] | [145-170] |
|  | A | [33-39) | [33-39) | [119-145) |
|  | B | [28-33) | [22-33) | [100-119) |
|  | C | <28 | <22 | <100 |
| Challenge Mode – 4% Taper | S | [39-48] | [40-48.6] | [140-170] |
|  | A | [33-39) | [33-40) | [115-140) |
|  | B  C | [28-33)  <28 | [28-33)  <28 | [90-115)  <90 |
| Challenge Mode – Open Apex | S | [39-48] | [38-46] | [140-170] |
|  | A | [33-39) | [31-38) | [115-140) |
|  | B | [28-33) | [27-31) | [90-115) |
|  | C | <28 | <27 | <90 |
| Challenge Mode – Severe Curvature | S | [39-48] | [40-48.6] | [140-170] |
|  | A | [33-39) | [33-40) | [115-140) |
|  | B | [28-33) | [28-33) | [90-115) |
|  | C | <28 | <28 | <90 |
| Challenge Mode – Severe Infection | S | [39-48] | [49-59] | [215-255] |
|  | A | [33-39) | [40-49) | [175-215) |
|  | B | [28-33) | [31-40) | [150-175) |
|  | C | <28 | <31 | <150 |
| Expert Mode | S | [41-48] | [53-59] | [220-255] |
|  | A | [36-41) | [47-53) | [185-220) |
|  | B | [28-36) | [35-47) | [160-185) |
|  | C | <28 | <35 | <160 |

**3.4 Final grade weighted score**

The final comprehensive score is obtained by summing the weighted points across all stages. The overall performance rating is then determined according to the criteria in **Supplementary Table 3**.

**Supplementary Table 3. Final grade weighted score**

| **Final Grade** | **Total Weighted Score** |
| --- | --- |
| S | SSS = 24; SS = [17–24); S = [13–17) |
| A | [8 -13) |
| B | [6 -8) |
| C | <6 |


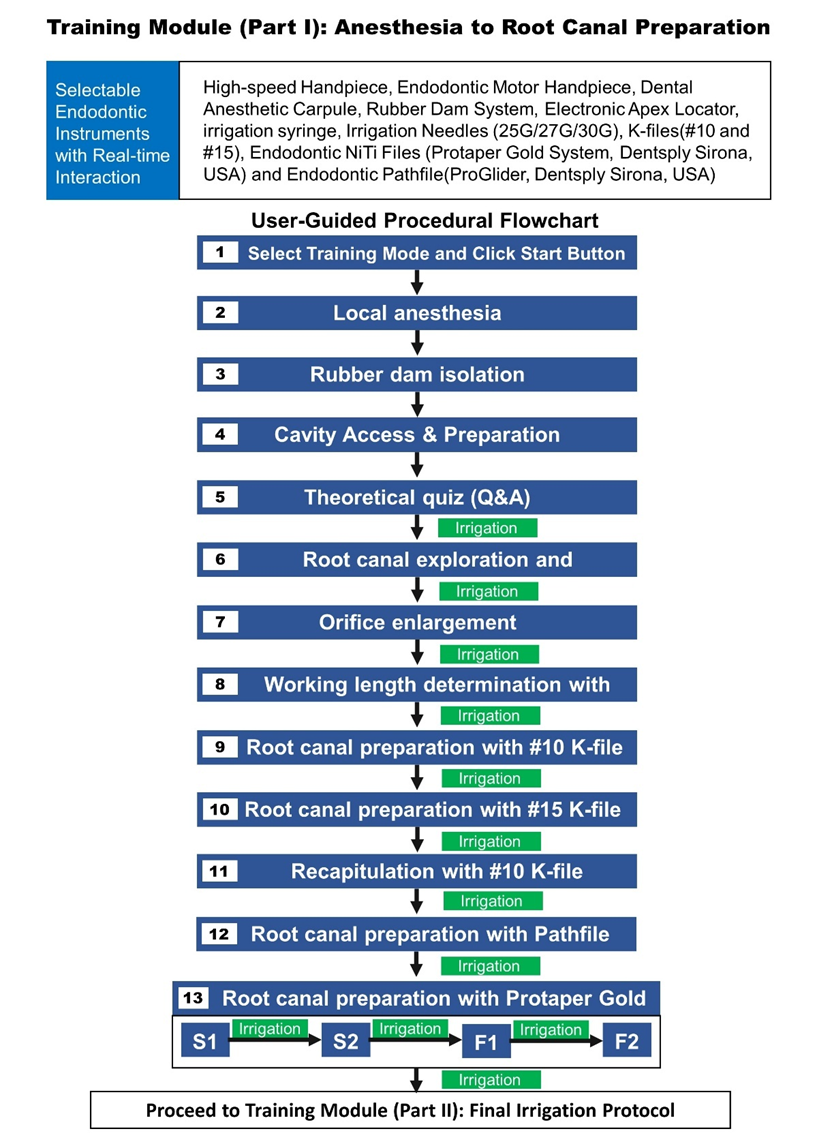


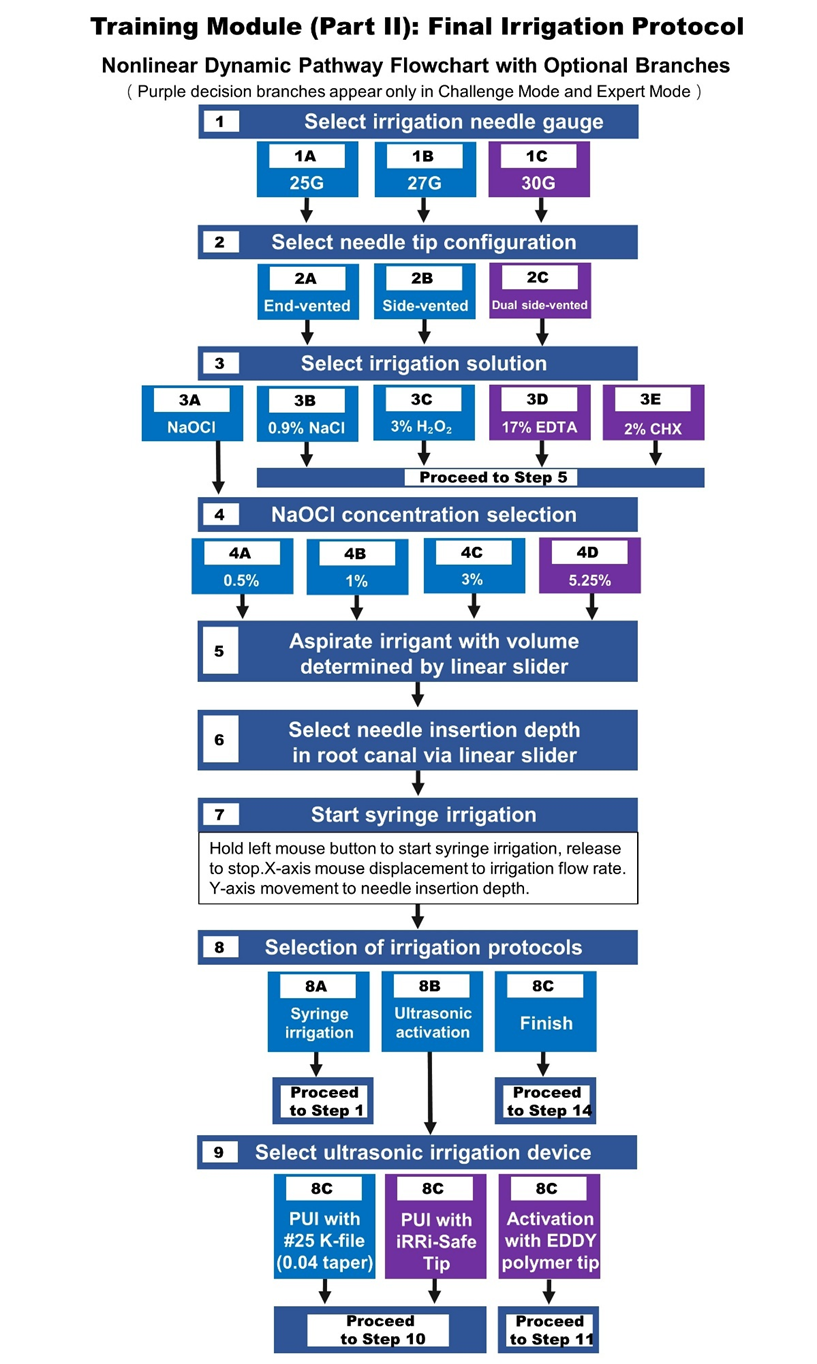


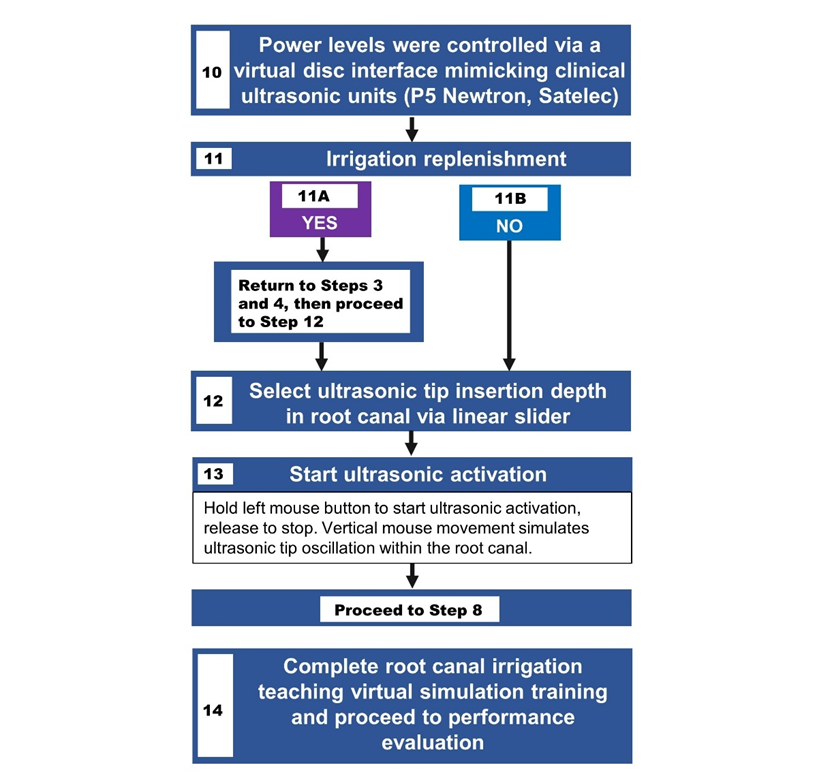


Supplementary Figure 1. Operational Flowchart of Root Canal Irrigation Teaching Virtual Simulation System


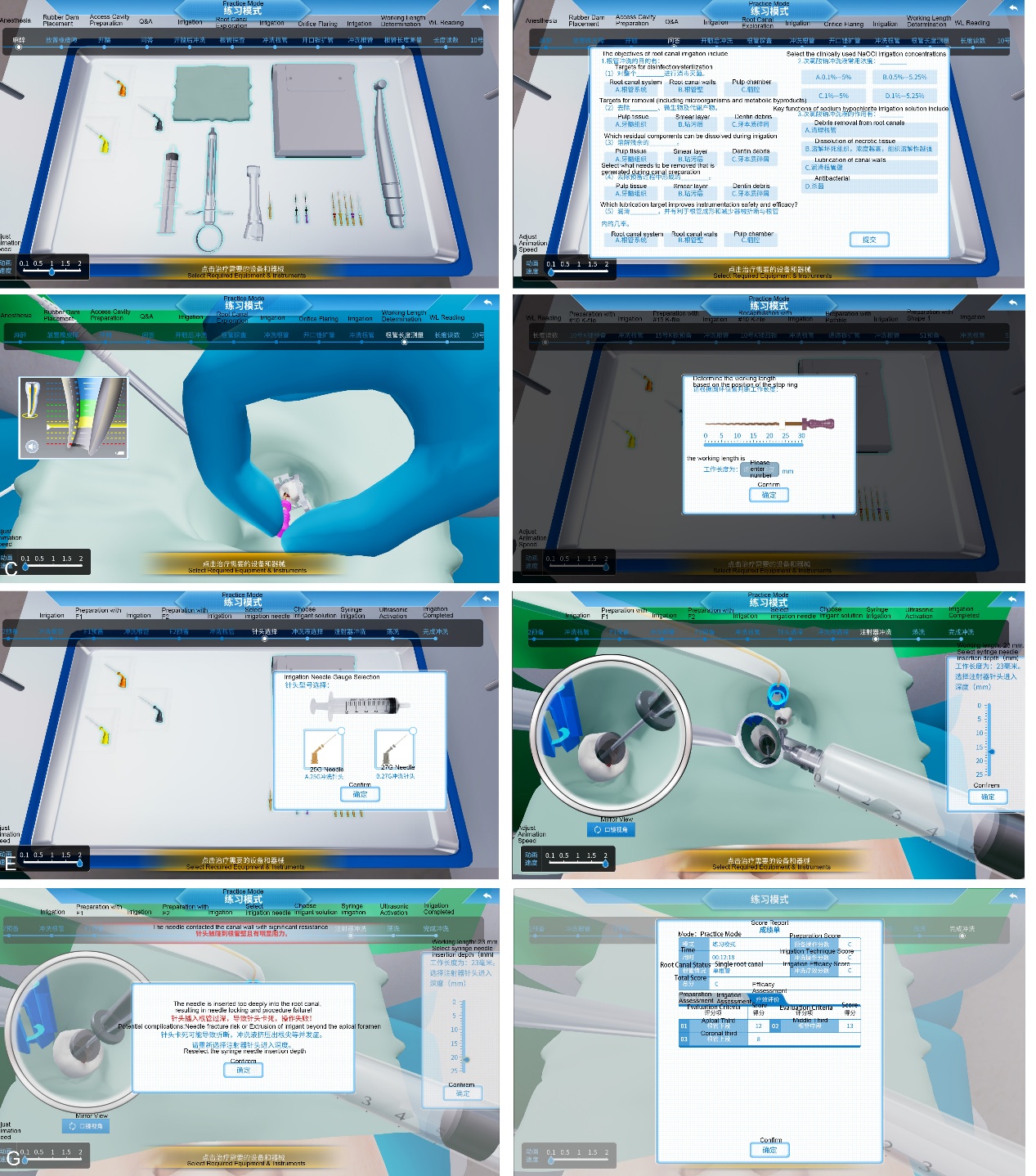


Supplementary Figure 2. Key interactive interfaces of the virtual simulation system for root canal irrigation.

A. Interface for instrument selection during canal preparation;

B. Interface for theoretical quiz assessment;

C. Simulation interface for working length determination using an apex locator;

D. Interface for recording and entering the measured working length;

E. Interface for selecting irrigation needle type and configuration;

F. Interface for selecting insertion depth of the irrigation device;

G. Error message interface triggered during final irrigation (e.g., tip binding or extrusion);

H. Interface displaying final performance scores and evaluation metrics.


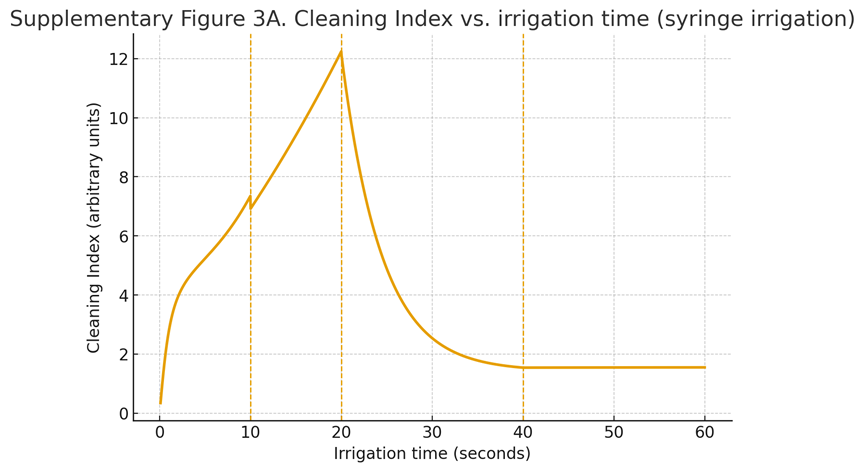


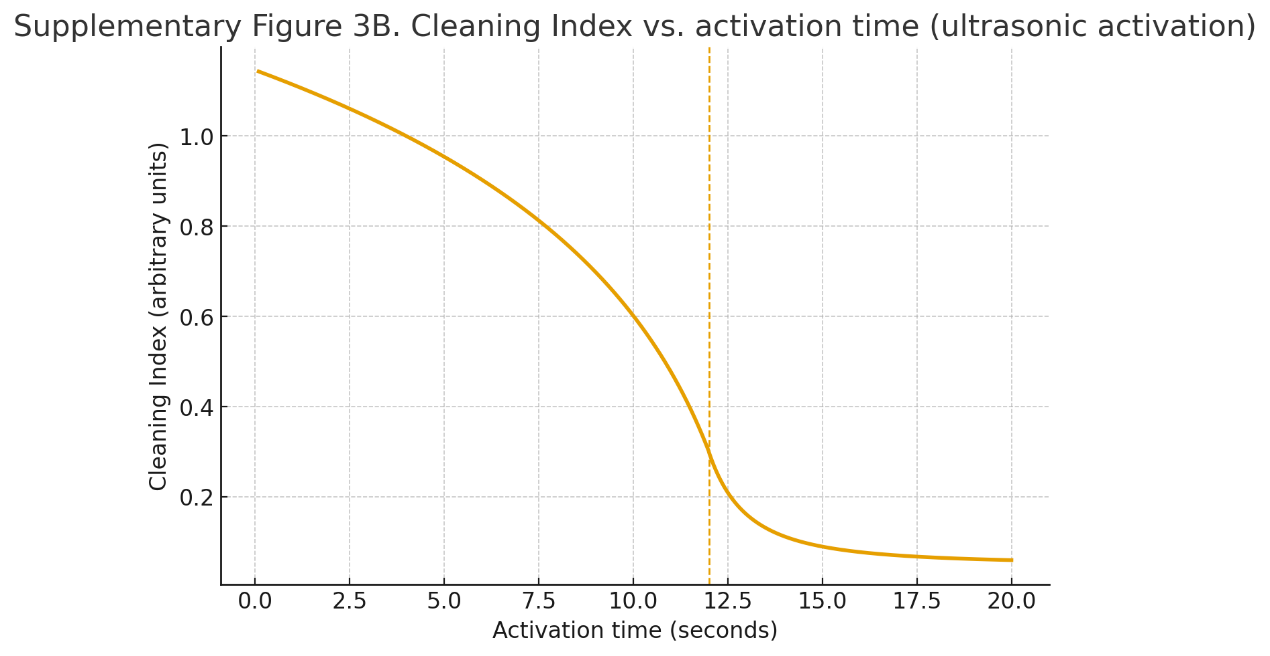


Supplementary Figure 3. Cleaning Index algorithm models.

(A) Cleaning Index as a function of irrigation time during syringe irrigation at a maximum flow rate of 0.5 mL/s.

(B) Cleaning Index as a function of activation time during ultrasonic activation.
