# Supplementary material for: Evaluation of a virtual simulation system for root canal irrigation training in preclinical dental education
Source: BMC Med Educ. 2025 Dec 3;26:27. doi: 10.1186/s12909-025-08387-x (PMC12781241; doi:10.1186/s12909-025-08387-x)
Supplement: Supplementary file 2 — Supplementary Material 2. Additional files 2: Scoring criteria and raw test results. [file 12909_2025_8387_MOESM2_ESM.docx]

**Addition File 2: Scoring criteria and raw test results**

The evaluation metrics in this study include theoretical test scores (S1 and S2), practical test scores (T1 and T2), and the automated score from the virtual simulation system (V1).

**1. Theoretical assessment (S1, S2)**

S1 and S2 represent scores on written theoretical tests focusing on root canal irrigation knowledge. Each test comprises 8 single-best-answer multiple-choice questions, with one point awarded per correct response. The maximum score is 8, and no penalties are applied for incorrect answers.

**2. Practical skill assessment (T1, T2)**

T1 and T2 refer to the pre- and post-training performance scores, respectively, each composed of two subcomponents.

**2.1 Operational Procedure Score**

Based on student performance on 3D-printed tooth models, evaluating:

(1) completeness of procedural steps;

(2) correct selection of instruments and irrigants;

(3) adherence to standardized protocols;

(4) appropriate irrigant volume and frequency;

(5) overall operational fluency.

**2.2 Irrigation Efficacy Score**

This score was determined through microscopic evaluation of residual debris on canal walls and in apical regions after the procedure. Each subcomponent was graded on a four-level ordinal scale: S = 8 points, A = 4 points, B = 2 points, and C = 1 point. The total practical score was the sum of both subcomponent scores, with a maximum possible score of 16.

**3. Virtual Simulation Score (V1)**

V1 denotes the automated score generated in examination mode of the Virtual Simulation Training System for Root Canal Irrigation. The algorithm encompasses procedural accuracy, instrument and irrigant selection, and final irrigation efficacy. A detailed description of the scoring algorithm is provided in **Additional file 1**.

**4. Result**

Individual test scores for all participants are summarized in **Supplementary Table 1**. Participants 1–26 were undergraduate students, and participants 27–34 were postgraduate students.

**Supplementary Table 1. Raw data table of individual test scores (n = 34)**

| **Participant** | **T1** **(pre-training practical score)** | **T2** **(post-training practical score)** | **V1** **(virtual simulation score)** | **S1** **(pre-training theoretical score)** | **S2** **(post-training theoretical score** |
| --- | --- | --- | --- | --- | --- |
| 1 | 6 | 8 | 10 | 7 | 8 |
| 2 | 4 | 12 | 8 | 6 | 8 |
| 3 | 5 | 6 | 5 | 6 | 7 |
| 4 | 3 | 4 | 5 | 5 | 6 |
| 5 | 6 | 6 | 10 | 6 | 8 |
| 6 | 4 | 8 | 10 | 6 | 6 |
| 7 | 8 | 12 | 10 | 7 | 8 |
| 8 | 4 | 8 | 6 | 5 | 7 |
| 9 | 4 | 6 | 5 | 7 | 7 |
| 10 | 2 | 4 | 5 | 4 | 5 |
| 11 | 8 | 12 | 12 | 8 | 7 |
| 12 | 3 | 6 | 6 | 4 | 6 |
| 13 | 12 | 16 | 20 | 8 | 8 |
| 14 | 8 | 12 | 12 | 6 | 8 |
| 15 | 6 | 8 | 10 | 7 | 7 |
| 16 | 4 | 8 | 8 | 7 | 7 |
| 17 | 12 | 8 | 12 | 7 | 8 |
| 18 | 4 | 6 | 8 | 7 | 7 |
| 19 | 6 | 8 | 6 | 6 | 7 |
| 20 | 16 | 16 | 20 | 8 | 8 |
| 21 | 10 | 8 | 8 | 5 | 6 |
| 22 | 6 | 12 | 12 | 6 | 8 |
| 23 | 3 | 8 | 10 | 6 | 8 |
| 24 | 2 | 4 | 4 | 6 | 8 |
| 25 | 4 | 6 | 4 | 7 | 8 |
| 26 | 3 | 8 | 12 | 5 | 7 |
| 27 | 4 | 8 | 12 | 8 | 8 |
| 28 | 6 | 12 | 16 | 7 | 7 |
| 29 | 8 | 16 | 16 | 7 | 8 |
| 30 | 6 | 6 | 10 | 8 | 6 |
| 31 | 3 | 8 | 10 | 5 | 7 |
| 32 | 8 | 12 | 12 | 7 | 8 |
| 33 | 2 | 6 | 5 | 6 | 6 |
| 34 | 12 | 12 | 10 | 8 | 8 |
