# Supplementary material for: Evaluation of a virtual simulation system for root canal irrigation training in preclinical dental education
Source: BMC Med Educ. 2025 Dec 3;26:27. doi: 10.1186/s12909-025-08387-x (PMC12781241; doi:10.1186/s12909-025-08387-x)
Supplement: Supplementary file 3 — Supplementary Material 3. Additional files 3: Frequency distribution of questionnaire responses. [file 12909_2025_8387_MOESM3_ESM.docx]

**Addition File 3: Frequency distribution of questionnaire responses**

*Scale: 1 = Strongly Disagree, 2 = Disagree, 3 = Neutral, 4 = Agree, 5 = Strongly Agree*

*Group: U= Undergraduate, P= Postgraduate, T= Total*

**Supplementary Table 1. Frequency distribution of questionnaire responses (n = 34)**

| Item and Statement | Group | 1 (%) | 2 (%) | 3 (%) | 4 (%) | 5 (%) |
| --- | --- | --- | --- | --- | --- | --- |
| 1. The system helped me understand the basic procedures and steps of root canal irrigation. | U | 0 | 3.80 | 11.50 | 26.90 | 57.70 |
|  | P | 0 | 0 | 12.50 | 25.00 | 62.50 |
|  | T | 0 | 2.90 | 11.80 | 26.50 | 58.80 |
| 2. I gained a clearer understanding of the selection and properties of irrigants. | U | 0 | 0 | 15.40 | 38.50 | 46.20 |
|  | P | 0 | 0 | 12.50 | 37.50 | 50.00 |
|  | T | 0 | 0 | 14.70 | 38.20 | 47.10 |
| 3. I was able to practice and master operational details such as dosage control and needle selection. | U | 0 | 0 | 7.70 | 34.60 | 57.70 |
|  | P | 0 | 0 | 12.50 | 37.50 | 50.00 |
|  | T | 0 | 0 | 8.80 | 35.30 | 55.90 |
| 4. I understood the importance and protocols of infection control during irrigation. | U | 0 | 0 | 7.70 | 26.90 | 65.40 |
|  | P | 0 | 0 | 12.50 | 37.50 | 50.00 |
|  | T | 0 | 0 | 8.80 | 29.40 | 61.80 |
| 5. Simulation-based training improved my confidence in performing actual procedures | U | 0 | 7.70 | 19.20 | 30.80 | 42.30 |
|  | P | 0 | 0 | 25.00 | 37.50 | 37.50 |
|  | T | 0 | 5.90 | 20.60 | 32.40 | 41.20 |
| 6. I realized it is difficult to achieve optimal outcomes in root canal irrigation. | U | 0 | 0 | 11.50 | 34.60 | 53.80 |
|  | P | 0 | 0 | 0 | 37.50 | 62.50 |
|  | T | 0 | 0 | 8.80 | 35.30 | 55.90 |
| 7. The system helped me understand and identify common complications of root canal irrigation (e.g., irrigant extrusion) and preventive measures. | U | 7.70 | 23.10 | 15.40 | 23.10 | 30.80 |
|  | P | 0 | 12.50 | 12.50 | 37.50 | 37.50 |
|  | T | 5.90 | 20.60 | 14.70 | 26.50 | 32.40 |
| 8. The sagittal view during irrigation helped me intuitively understand the internal canal anatomy and cleaning steps. | U | 0 | 3.80 | 7.70 | 30.80 | 57.70 |
|  | P | 0 | 0 | 12.50 | 25.00 | 62.50 |
|  | T | 0 | 2.90 | 8.80 | 29.40 | 58.80 |
| 9. Simulation-based learning improved my spatial understanding and operational accuracy during irrigation. | U | 0 | 7.70 | 11.50 | 30.80 | 50.00 |
|  | P | 0 | 0 | 12.50 | 37.50 | 50.00 |
|  | T | 0 | 5.90 | 11.80 | 32.40 | 50.00 |
| 10. The user interface was simple and intuitive to operate. | U | 0 | 0 | 3.80 | 30.80 | 65.40 |
|  | P | 0 | 0 | 0 | 37.50 | 62.50 |
|  | T | 0 | 0 | 2.90 | 32.40 | 64.70 |
| 11. The instructional design was reasonable and consistent with real clinical procedures. | U | 0 | 0 | 3.80 | 26.90 | 69.20 |
|  | P | 0 | 0 | 0 | 25.00 | 75.00 |
|  | T | 0 | 0 | 2.90 | 26.50 | 70.60 |
| 12. Visual and animation effects made the root canal changes more intuitive. | U | 0 | 0 | 7.70 | 26.90 | 65.40 |
|  | P | 0 | 0 | 12.50 | 25.00 | 62.50 |
|  | T | 0 | 0 | 8.80 | 26.50 | 64.70 |
| 13. Error prompts and operational guidance in the system enhanced my understanding of irrigation protocols. | U | 0 | 3.80 | 11.50 | 30.80 | 53.80 |
|  | P | 0 | 0 | 12.50 | 37.50 | 50.00 |
|  | T | 0 | 2.90 | 11.80 | 32.40 | 52.90 |
| 14. The operation ran smoothly without significant lag or delays. | U | 0 | 0 | 11.50 | 34.60 | 53.80 |
|  | P | 0 | 0 | 0 | 37.50 | 62.50 |
|  | T | 0 | 0 | 8.80 | 35.30 | 55.90 |
| 15. My hand movements during irrigation matched well with the system’s feedback. | U | 0 | 7.70 | 15.40 | 30.80 | 46.20 |
|  | P | 0 | 0 | 12.50 | 37.50 | 50.00 |
|  | T | 0 | 5.90 | 14.70 | 32.40 | 47.10 |
| 16. The virtual feedback (e.g., positional changes) helped me understand the effects of my actions. | U | 0 | 3.80 | 11.50 | 30.80 | 53.80 |
|  | P | 0 | 0 | 0 | 50.00 | 50.00 |
|  | T | 0 | 2.90 | 8.80 | 35.30 | 52.90 |
| 17. I feel that my root canal irrigation skills improved after using the system. | U | 0 | 0 | 11.50 | 30.80 | 57.70 |
|  | P | 0 | 0 | 0 | 37.50 | 62.50 |
|  | T | 0 | 0 | 8.80 | 32.40 | 58.80 |
| 18. The step-by-step feedback and scoring helped me identify and improve my weaknesses. | U | 0 | 0 | 7.70 | 34.60 | 57.70 |
|  | P | 0 | 0 | 0 | 50.00 | 50.00 |
|  | T | 0 | 0 | 5.90 | 38.20 | 55.90 |
| 19. The system effectively guided me in selecting needles, irrigants, and techniques. | U | 0 | 0 | 11.50 | 30.80 | 57.70 |
|  | P | 0 | 0 | 12.50 | 25.00 | 62.50 |
|  | T | 0 | 0 | 11.80 | 29.40 | 58.80 |
| 20. The difficulty design between practice and test modes helped improve my skills progressively. | U | 0 | 3.80 | 7.70 | 26.90 | 61.50 |
|  | P | 0 | 0 | 0 | 37.50 | 62.50 |
|  | T | 0 | 2.90 | 5.90 | 29.40 | 61.80 |
| 21. The difficulty levels in the system reflect real clinical problems. | U | 0 | 0 | 15.40 | 23.10 | 61.50 |
|  | P | 0 | 0 | 12.50 | 25.00 | 62.50 |
|  | T | 0 | 0 | 14.70 | 23.50 | 61.80 |
| 22. After using the system, I gained a visual understanding of the function and outcomes of irrigation. | U | 0 | 0 | 3.80 | 30.80 | 65.40 |
|  | P | 0 | 0 | 0 | 37.50 | 62.50 |
|  | T | 0 | 0 | 2.90 | 32.40 | 64.70 |
| 23. The system made me realize the need to strengthen irrigation procedures during clinical operations. | U | 0 | 0 | 3.80 | 26.90 | 69.20 |
|  | P | 0 | 0 | 0 | 25.00 | 75.00 |
|  | T | 0 | 0 | 2.90 | 26.50 | 70.60 |
| 24. I am satisfied with the overall effectiveness of the root canal irrigation simulation system. | U | 0 | 0 | 3.80 | 34.60 | 61.50 |
|  | P | 0 | 0 | 0 | 37.50 | 62.50 |
|  | T | 0 | 0 | 2.90 | 35.30 | 61.80 |
| 25. I am willing to continue using the system for further study. | U | 0 | 0 | 3.80 | 26.90 | 69.20 |
|  | P | 0 | 0 | 0 | 25.00 | 75.00 |
|  | T | 0 | 0 | 2.90 | 26.50 | 70.60 |
| 26. I would recommend this system to other students or clinicians for learning root canal irrigation. | U | 0 | 0 | 3.80 | 30.80 | 65.40 |
|  | P | 0 | 0 | 0 | 37.50 | 62.50 |
|  | T | 0 | 0 | 2.90 | 32.40 | 64.70 |
